# Supplementary material for: Splice-Junction-Based Mapping of Alternative Isoforms in the Human Proteome
Source: Cell Rep. Author manuscript; Available in PMC 2020 Jan 15. (PMC6961840; doi:10.1016/j.celrep.2019.11.026)
Supplement: 2 [file NIHMS1546469-supplement-2.zip › DF1/PXD006675/CardiacFibroblast/CardiacFibroblast_22_HMG20B_ALGSAAAAPAGGK.pdf]

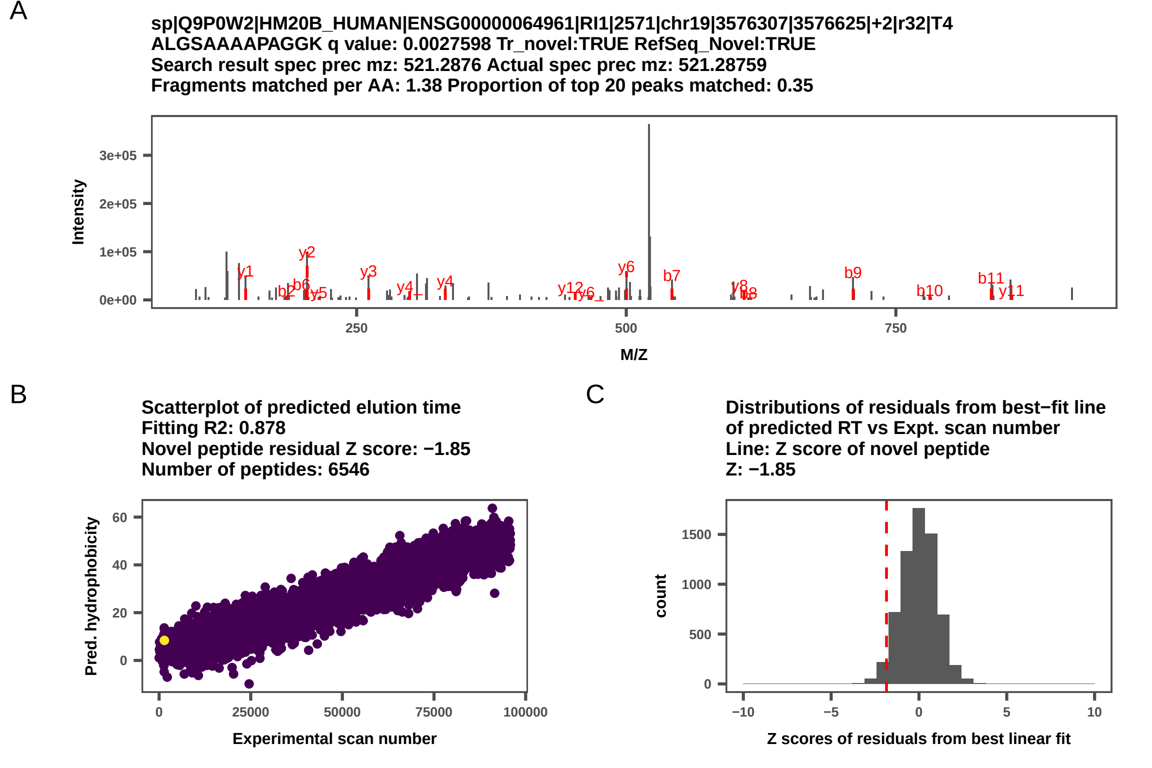
A
sp|Q9P0W2|HM20B_HUMAN|ENSG00000064961|RI1|2571|chr19|3576307|3576625|+2|r32|T4
ALGSAAAAPAGGK q value: 0.0027598 Tr_novel:TRUE RefSeq_Novel:TRUE
Search result spec prec mz: 521.2876 Actual spec prec mz: 521.28759
Fragments matched per AA: 1.38 Proportion of top 20 peaks matched: 0.35
Intensity
3e+05
2e+05
1e+05
0e+00
y1
y2
b2
b6
y5
y3
y4
y4
y12
y6
y6
b7
y8
b8
b9
b10
b11
y11
250
500
750
M/Z
B
Scatterplot of predicted elution time
Fitting R2: 0.878
Novel peptide residual Z score: −1.85
Number of peptides: 6546
Pred. hydrophobicity
60
40
20
0
0
25000
50000
75000
100000
Experimental scan number
C
Distributions of residuals from best−fit line
of predicted RT vs Expt. scan number
Line: Z score of novel peptide
Z: −1.85
count
1500
1000
500
0
−10
−5
0
5
10
Z scores of residuals from best linear fit
